# The Relationship between General Movements and Risk Factors in Moderate-Late Preterm Infants: A Prospective Cohort Study

**DOI:** 10.3390/jcm12247763

**Published:** 2023-12-18

**Authors:** Javier Merino-Andrés, Soraya Pérez-Nombela, Álvaro Hidalgo-Robles, María del Prado Pérez-Domínguez, Lorena Prieto-Sánchez, Francisco Javier Fernández-Rego

**Affiliations:** 1 Faculty of Physiotherapy and Nursing, Universidad de Castilla-La Mancha, Avenida de Carlos III s/n, 45071 Toledo, Spain; javier.merino@uclm.es; 2 Physiotherapy Research Group of Toledo (GIFTO), Universidad de Castilla-La Mancha, Avenida de Carlos III s/n, 45071 Toledo, Spain; 3 Centro Crecer, 45007 Toledo, Spain; 4 Faculty of Education, International University of La Rioja, 26006 Logroño, Spain; alvaro.hidalgo@unir.net; 5 Hospital General Universitario Nuestra Señora del Prado, 45600 Talavera de la Reina, Spain; mdelp@sescam.jccm.es (M.d.P.P.-D.); lprieto@sescam.jccm.es (L.P.-S.); 6 Physiotherapy Department, University of Murcia, 30100 Murcia, Spain; fjfernan@um.es; 7 Early Care Research Group (GIAT), University of Murcia, 30100 Murcia, Spain

**Keywords:** general movement assessment, Nursery Neurobiologic Risk Score, Perinatal Risk Inventory, moderate-late preterm, neurodevelopmental conditions

## Abstract

Introduction: Moderate-late preterm infants constitute the largest segment of preterm births globally. While previously considered to have a low neurological risk, recent research has uncovered an elevated incidence of neurodevelopmental conditions in this group. This study aimed to assess the relationship between the general movement assessment and birth-related risk factor-based tools in moderate-late preterm infants. Methods: A prospective cohort study of 65 moderate-late preterm infants in a neonatal intensive care unit involved the evaluation of general movements, the Nursery Neurobiologic Risk Score, and the Perinatal Risk Inventory. Associations were analyzed using Fisher’s exact test, Spearman’s correlation was used for ordinal variables, and backward stepwise logistic regression was used to identify predictor variables for the assessments. Results: The findings indicated a high prevalence of normal (41%) and poor (52%) repertoire patterns during the writhing period. While no significant associations were found between the three assessments, a slight approximation emerged between dysmorphic traits and patterns (*p* = 0.053). Furthermore, an extended period of ventilation correlated with a higher likelihood of developing a cramped synchronized pattern and there was a correlation between both risk factor-based tools (*p* < 0.001). Conclusions: This research enhances our understanding of the early impact on general movement assessments in moderate-late preterm infants. While no clear relationship emerged between general movement assessment and risk factor-based tools, there was a subtle connection noted with dysmorphic traits. A longer ventilation duration was linked to a higher risk of developing cramped synchronized patterns.

## 1. Introduction

Moderate-late preterm infants (MLPTs) born between 32^0/7^ and 36^6/7^ gestational weeks represent the largest group of preterm births worldwide, accounting for 84.3% of preterm births in 2010 [1] and 84.7% in 2014 [2]. Although they were previously considered to have a very low neurological risk, recent research has revealed a higher incidence of neurodevelopmental conditions when compared to full-term infants [2]. This specific group of preterm infants (PTs) more frequently presents nonneurological signs, symptoms, and medical diagnoses, with hyperbilirubinemia, hypoglycemia, and respiratory alterations being among the most prevalent [3,4,5]. These conditions increase the risk of developing long-term intellectual disabilities, neuropsychiatric conditions, and asthma [4].

Given the complexity of clinical conditions within the MLPT population, the early detection of infants at high risk for neurodevelopment issues is essential for providing timely diagnostic prognoses. The work of Novak et al. in 2017 marked a significant milestone in understanding and implementing the high risk of cerebral palsy [6]. This was achieved through the use of three highly predictive tools applicable before five months of age: magnetic resonance imaging (MRI) (86–89% sensitivity [6] and 89–97% specificity [7]), the qualitative assessment of Prechtl’s general movements (GMA) (98% sensitivity [6] and 91% specificity [7]), and the Hammersmith Infant Neurological Examination (HINE) (90% sensitivity [6] and 90% specificity [8]). These assessments are recommended for infants within various categories, including PTs, infants diagnosed with neonatal encephalopathy, historical or neurological risk factors (such as low gestational age, congenital malformations, or delayed intrauterine growth), risk factors identified by parents or primary caregivers, and red flags in motor development, such as the inability to sit independently by nine months of corrected age and asymmetric use of manual function [6].

In this context, clinical history plays a pivotal role in initiating the entire screening process from the earliest stages of life. Consequently, there are instruments designed to complement the administration of general movements (GMs), HINE, and MRI, such as the Nursery Neurobiologic Risk Score (NBRS) [9] and the Perinatal Risk Inventory (PERI) [10]. These instruments were developed based on an assessment of the different risk factors that newborns may encounter during their stay in a neonatal intensive care unit (NICU). The NBRS is administered during a PT’s NICU stay, while the PERI is conducted after the child is discharged from the service. Both tests have been examined and compared to determine their effectiveness in predicting high risk in newborns, as demonstrated in the study by Zaramella et al. in 2008 [11]. The findings confirm the predictive power of both instruments for identifying high-risk cases, with no noticeable differences between them [11].

Due to the potential long-term repercussions on MLPT neurodevelopment, such as cognitive deficits, learning difficulties, or behavioral alterations [12], surveillance of this population group is recommended during the first two years of life to promptly detect any possible issues. It is estimated that 30% of late PTs are expected to require various services for early intervention [13].

Therefore, the primary objective of this study was to evaluate the relationship between GMs, which offer the highest sensitivity for detecting cerebral palsy, and instruments based on birth-related risk factors (NBRS and PERI) within the MLPT population. The secondary objectives included determining the frequency of different GM patterns during the writhing period, assessing the relationship between the NBRS and PERI within the MLPT population, and evaluating the relationship between the assessment tools and attendance at early intervention services.

## 2. Methods

### 2.1. Design

This prospective cohort study aimed to investigate the relationship between GMA during the writhing period and risk factors for MLPTs during their stay in NICU and intermediate care. Ethical approval was obtained from the Clinical Research Ethics Committee of the Nuestra Señora del Prado Hospital in Talavera de la Reina (33/2018) and the Clinical Research Ethics Committee of the Virgen de la Salud Hospital of Toledo (426/2019). This study is registered in ClinicalTrials under registration number NCT04073836.

### 2.2. Participants

The subjects included in this study were MLPT infants (born between 32^0/7^ and 36^6/7^ gestational weeks) who were admitted to an NICU or intermediate care at either Nuestra Señora del Prado Hospital in Talavera de la Reina or the Virgen de la Salud Hospital in Toledo during the years 2018, 2019, 2020, and 2021. Participation in this study needed parental agreement through an informed consent form. Exclusion criteria encompassed MLPT infants who had been prescribed any of the following medications: barbiturates, antiepileptics, indomethacin, or dexamethasone.

### 2.3. Outcome Measures

The Prechtl GMA method is acknowledged for its objectivity, validity, and reliability in assessing the quality of GMs [14]. During term age and within the first 2 months post-term, GMs are commonly referred to as writhing movements [15]. They are characterized by small to moderate amplitudes and slow to moderate speeds [15]. Typically, they are elliptical in form, creating the impression of a writhing quality. Abnormal GMs before the third month can be classified into two categories: “poor repertoire” and “cramped synchronized” movements. A poor repertoire indicates monotonous sequences of successive movement components, lacking the complexity observed in normal GMs. Cramped synchronized GMs appear rigid and lack the smoothness and fluency characteristic of normal GMs, with limb and trunk muscles contracting and relaxing almost simultaneously [14].

The NBRS involves thirteen items that affect cell injury in the neonatal brain [9]. These items include the presence of an Apgar score, PaO_2_, ventilation, blood pH, apnea with bradycardia, hypotension, patent ductus arteriosus, seizures, intraventricular hemorrhage, periventricular leukomalacia, infection, hypoglycemia, and bilirubin levels throughout the entire admission period. Each item is assigned a score of 0 if absent or graded as 1, 2, or 4, representing a geometric progression of scores that weigh the severity or duration of an event more heavily. Scores are recorded during a newborn’s hospital stay before discharge, and the total score is the sum of these thirteen items [16]. Children were categorized into three risk levels as described by Brazy et al.: low (≤4), moderate (5 to 7), and high (≥8). A revised 2-week score of >5 or a discharge score of >6 demonstrated 100% specificity and had a 100% positive predictive value for abnormal outcomes at 24 months of age [9].

The PERI items utilize an ordinal scale ranging from 0 to 3 to score items during a newborn’s hospital stay before discharge. This scale is designed so that a higher score corresponds to a greater risk of subsequent developmental abnormalities. Specific PERI items include the Apgar score, gestational age and infant weight appropriateness, infections, intraventricular hemorrhage, hydrocephalus, seizures, head growth (preterm or term newborn), dysmorphic traits, meningitis, electroencephalogram (EEG), brain scans, ventilation, polycythemia, hypoglycemia, hyperbilirubinemia, and long-term physical illnesses such as bronchopulmonary dysplasia (BPD). Both NBRS and PERI scores provide a comprehensive assessment that covers the entire duration of a hospital stay and can be determined at the time of discharge. For these scores, cutoff scores of ≥6 for NBRS and ≥10 for PERI have been established to identify most infants with abnormal outcomes [9,10]. Table S1 shows the scoring criteria for the Nursery Neurobiologic Risk Score and Table S2 shows the scoring criteria for the Perinatal Risk Inventory.

### 2.4. Procedure

To assess GMs in PTs, we recorded 10 min of video during their stay in NICU or intermediate care. We positioned a GoPro Hero 7™ camera at the foot of cribs on a tripod, capturing each subject in a supine position. These video recordings were independently reviewed by two assessors (M C-M and J M-A). In instances where discrepancies arose between their assessments, a third assessor (Á H-R) was responsible for making the final determination. All three assessors had previously either basic or advanced certification in the Prechtl GMA method.

The administration of PERI and NBRS involved monitoring the clinical history and discharge reports from the neonatology service for each subject. An assessor (MP P-D) who was blinded to the GMA results conducted this process. The same assessor also reviewed the medical history of each subject at two years of corrected age to confirm the variables related to the medical diagnosis and their inclusion in any early intervention program.

### 2.5. Statistics

For the demographic variables, we conducted a descriptive analysis using percentages and frequencies. To assess homogeneity, a chi-square test was performed. In addition, we employed Fisher’s exact test to analyze different categorical variables for potential associations. When examining quantitative variables, a one-factor ANOVA study was carried out once the assumptions of normality and homoscedasticity had been verified. In all our analyses, the significance value was set at *p* < 0.05. To explore the correlation between ordinal variables, Spearman’s correlation coefficient was used, with a positive value indicating a direct relationship, a negative value indicating an inverse relationship, and a value of zero indicating no relationship.

Furthermore, a backward stepwise logistic regression was performed to identify potential predictor variables for the different GM patterns and PERI and NBRS items. This allowed us to determine which risk factors might exert a more significant impact on GM patterns within the MLPT population. In each step of this regression, the *p* value was used as the criterion for variable selection, and the Akaike information criterion converged. All analyses were performed with R version 4.0.3 (R Core Team 2020).

## 3. Results

### 3.1. Demographics

Within the recruitment stage, 216 MLPTs and their families were informed. Ultimately, 65 infants (30.10%) were included. Reasons for exclusion were inability to record due to unstable clinical conditions (*n* = 9, 4.16%), absence of informed consent from parents or main caregivers (*n* = 60, 27.77%), transfer to another hospital (*n* = 2, 0.92%), and hospitalization during the COVID-19 restrictions (*n* = 80, 37.05%). Patient characteristics are shown in Table 1.

### 3.2. Relationship between Outcome Measures

The distribution of subjects across different GM patterns during the writhing period was examined. Normal patterns were observed for 41.55% (*n* = 27), poor repertoire patterns were observed for 52.30% (*n* = 34), and cramped synchronized patterns were observed for 6.15% (*n* = 4), while no results were obtained for chaotic patterns. When we considered the distribution by week of gestation, we observed differences between the moderate PT (*n* = 16) and late PT (*n* = 49) groups. In the moderate PT group, 50% exhibited normal patterns, 43.75% exhibited poor repertoire patterns, and 6.25% fell into the cramped synchronized category. On the other hand, in the late PT population, 36.73% exhibited normal patterns, 57.14% exhibited poor repertoire patterns, and 6.12% exhibited cramped synchronized patterns.

Regarding the referral to early intervention services, 7.40% of infants with normal patterns (*n* = 2) received early intervention. In contrast, 26.47% of those with poor repertoire patterns (*n* = 9) received it, while 25% of infants with cramped synchronization (*n* = 1) were referred to it.

We examined the association between the different patterns of GMs and the risk factor assessment tools. No significant association between the PERI total score and GMs was found (*p* > 0.05), except for a small approximation without statistical significance with the item related to dysmorphic traits (*p* = 0.053). Similarly, when assessing the relationship between GM patterns and the total NBRS score, we obtained no significant association between the two tools (*p* > 0.05).

**Figure 1:**
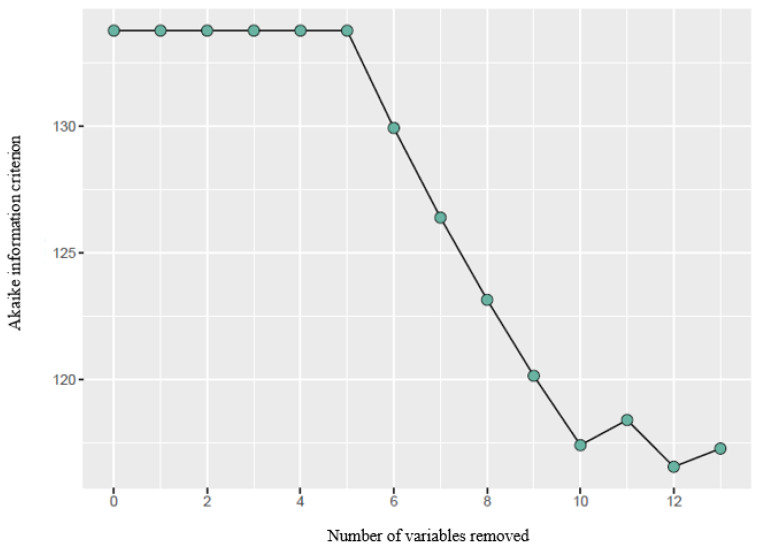
Progression of backward stepwise regression using the Akaike information criterion, which indicates the best predictive model by eliminating variables.

**Figure 2:**
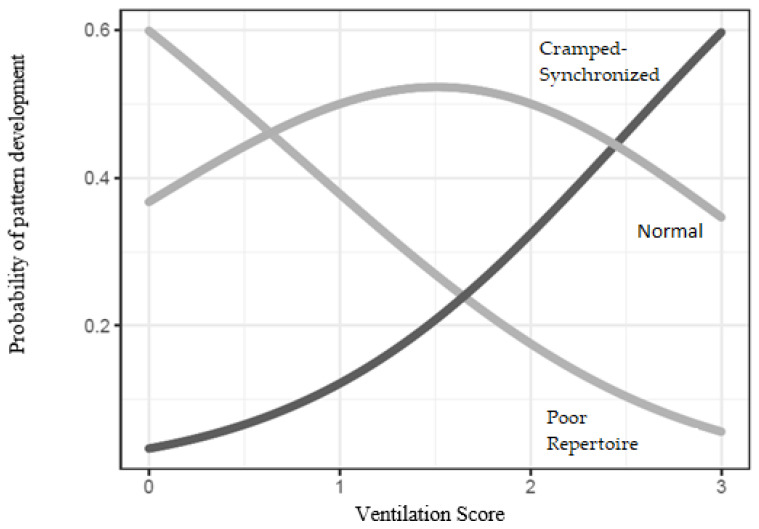
Correlation between ventilation and general movements assessment. A higher ventilation score (more days of ventilation use) was associated with an increased risk of cramped synchronized patterns.

After performing backward stepwise logistic regression (Figure 1), a direct relationship was found between increased exposure to ventilation during NICU stay and the development of cramped synchronized patterns (Figure 2).

**Figure 3:**
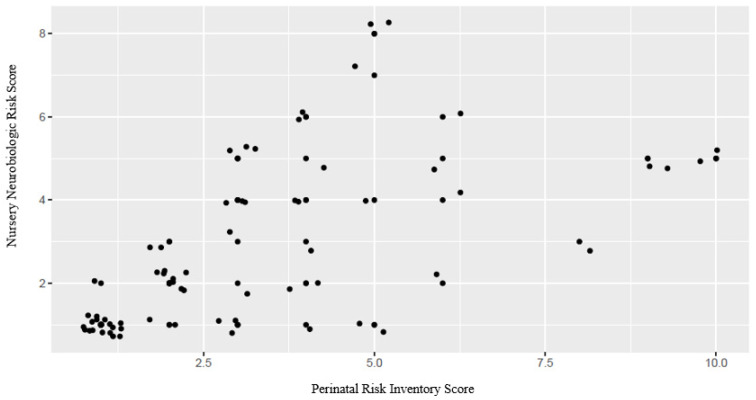
Correlation between the Nursery Neurobiologic Risk Score and Perinatal Risk Inventory. A positive correlation can be observed between both assessment tools.

Finally, when correlating the PERI and NBRS, we found a significant correlation (*p* < 0.001), indicating a relationship between both instruments. Specifically, higher PERI score levels corresponded to higher NBRS risk scores, and negative results demonstrated an inverse relationship for the risk factors of both instruments (Figure 3 and Table 2). Neither of the two tools showed a direct relationship with access to early intervention services (*p* > 0.05).

## 4. Discussion

The findings of this study provide information on the prevalence of different GM patterns within the MLPT population. Consistent with other studies, we observed a higher percentage of normal and poor repertoire patterns. Brogna et al. in 2013 found a higher percentage of normal patterns (82%) than poor repertoire patterns (13%) [17]. In a large study by Einspieler et al. in 2015 focusing on the general movement optimality score, they reported a distribution for the moderate PT population, with 11.44% showing normal writhing, 71.04% showing a poor repertoire, and 18.67% showing cramped synchronized. For the late PT population, the distribution was 45.65% normal patterns, 28.26% poor repertoire, and 21.30% cramped synchronized [18].

Spittle et al. in 2016 in their study on the neurological and behavioral relationship during the first month of life in a population of newborns born between weeks 32 and 42 of gestation found a distribution for the moderate PT population of 25% normal patterns, 73% poor repertoire patterns, and 2% cramped synchronized. For the late PT group, it was 32% normal patterns, 68% poor repertoire, and 0% cramped synchronized [19]. The variations in the results between our study and prior research may be attributed to the diverse risk factors present in each study’s subject population, as well as the distinct perinatal care provided during their NICU stay [20]. Consequently, it appears that the two predominant patterns observed in the MLPT population during the writhing period are normal and poor repertoire patterns.

In terms of the relationship between GMs and risk factor assessment tools, we found no significant association between the total scores of these tools and GMs during the writing period. However, a slight approximation was identified, particularly in the context of dysmorphic traits and GM patterns. Dysmorphic traits have been previously studied in association with congenital anomalies. Infants with these traits often undergo various surgical procedures during their time in the NICU and intermediate care. In 2015, Crowle et al. examined GMs during the writhing period in a population of newborns with congenital disorders who underwent surgical interventions. The emergence of poor repertoire patterns was the most frequent finding [21].

The absence of an association between GMs and risk factor assessment tools may be attributed to the specific morbidities and characteristics of the MLPT population. Compared to full-term infants, this particular population exhibits a heightened frequency of risk factors and morbidities associated with respiratory or circulatory failure, sepsis, hypoglycemia, hyperbilirubinemia, and eating problems [3,22,23]. These factors play a pivotal role in shaping the overall neurodevelopmental trajectory of each PT. It is conceivable that the full impact of these factors on neurodevelopment may only become apparent in the long term, particularly as the preterm population progresses into their preschool educational stage. Therefore, it is possible that the development of neurological conditions, such as cerebral palsy, may require not only a high number of these risk factors but also a combination of other neurological factors or prolonged exposure to those of metabolic origin.

Regarding the primary risk factors observed in MLPT individuals and their impact on GM assessment, hyperbilirubinemia has been studied by various authors. Soorani-Lunsing et al. in 2001 found a higher occurrence of abnormal patterns in the fidgety period, which indicated a minor neurological disorder within a cohort of full-term newborns [24]. Kahraman et al. in 2021 conducted a similar analysis in a population of full-term newborns, revealing low scores for the motor optimality score (GMA quantitative assessment), signifying a poor motor repertoire in their development [25]. Finally, in 2013, Lunsing et al. identified a higher frequency of abnormal patterns in the fidgety period. While statistical significance was not established when compared to the control group, a direct relationship was observed with the amount of pigment [26]. Consequently, there may be a direct connection between bilirubin levels and potential GM alterations. A systematic review of the effects of bilirubin on neurodevelopment emphasized that the use of GMs would be a valid tool to detect minor neurological disorders between 3 and 5 months of age. However, in this study where participants underwent interventions to reduce bilirubin when pathological levels were detected, no relationship was found between the variables related to bilirubin levels and the GM patterns during the writhing period [27].

In 2019, Nogolová et al. conducted a study that examined the impact of neonatal hypoglycemia on GMs. They found a high frequency of abnormal patterns, particularly a poor repertoire, during the writhing period. Notably, many of these abnormal patterns transitioned to normal patterns in the fidgety period. However, some patterns did not conform to typical movements, suggesting potential alterations in neurodevelopment. The authors highlighted the importance of monitoring GMs in both periods and recommended the use of other neurological assessment tools for early detection [28]. Regarding other nonneurological risk factors, Skworc et al. in 2020 analyzed the relationship between early or late infections and GMs in a broad population of PTs. In contrast to our study, their findings revealed a direct relationship between GMs and the occurrence of infections, particularly, a higher frequency of poor repertoire patterns during the first 14 days of life. The authors concluded that it is essential to monitor the PT population with a history of infections, emphasizing the importance of understanding their long-term neurodevelopment [29].

Exploring the influence of respiratory alterations, Hitzert et al. in 2014 conducted a study in a population of extremely PT infants at risk of developing BPD. Their findings revealed high percentages of abnormal GMs during the writhing period. As for hypoglycemia [28], they tended to normalize during the fidgety period [30].

The early detection and treatment of risk factors, as per clinical practice guidelines and intervention protocols, are crucial to mitigate their potential impact on brain development. Among the three most prevalent nonneurological risk factors in the MLPT population (hypoglycemia, hyperbilirubinemia, and respiratory disorders) [22,23,31,32], the precise thresholds that lead to brain damage are not yet known. For instance, in newborns with neonatal hypoglycemia, Rasmussen et al. in 2020 suggested that amounts below 30 mg/dL may affect fine motor skills but not necessarily other areas of neurodevelopment [33]. However, this finding contrasts with the results from McKinlay et al. and Kerstjens et al. [34,35] who observed long-term neurodevelopment alterations in the same population.

While extreme levels of bilirubin accumulation are well documented, the neurological consequences of moderate or mild pigment accumulation remain uncertain [36]. A similar lack of clarity is observed in respiratory disorders. The worst respiratory prognosis for PT infants, specifically BPD, has been linked to subsequent cerebral palsy development [37], particularly in cases where abnormal patterns in the fidgety period coincide with hyperbilirubinemia [38]. However, the repercussions of mild respiratory system alterations have not been precisely quantified, as indicated in this research. Notably, backward stepwise regression analysis revealed a compelling connection between the duration of ventilation during the hospital stay and the likelihood of developing cramped synchronized GMs.

In 2008, Zaramella et al. concluded that both the PERI and NBRS tools are reliable and valuable for assessing risk factors associated with the current or future severity of a disease [11]. These results align with the findings of our study, which also established a direct correlation between these risk factor assessment tools. In a study by Mehler et al. in 2014 that focused on MLPT infants and administered the NBRS to assess neurological risk, no significant results were obtained [39]. However, the use of these instruments based on risk factors can facilitate the clinical practices of the different professionals who intervene in this population as this would help to more continuously monitor those subjects with more critical scores and could also facilitate access to early intervention services based on the scores obtained with these instruments.

Our study found no direct relationship between abnormal GMs, PERI and NBRS scores, and the utilization of early intervention services. In this sense, only 20% of the subjects attended early intervention services. In contrast, a study by Chatzioanidis et al. in 2018 reported that 39.6% of a late PT population accessed early intervention services at six months of age, increasing to 57.54% by 12 months of age [12]. These numbers reflect the insufficient follow-up for the MLPT population, potentially leading to delayed detection and intervention. Such delays limit the window of opportunity for therapeutic interventions based on neuroplasticity to reduce the consequences of premature birth [12].

Therefore, continuous monitoring is imperative for MLPT infants with nonneurological risk factors as they often exhibit minor neurological disorders or face academic challenges starting in preschool [40]. Typically, more rigorous follow-ups are conducted for clinical conditions with greater neurological implications, such as encephalopathies or prematurity below the 32nd week of gestation [6]. However, the MLPT population may lack adequate follow-up, leaving the potential long-term repercussions on their neurodevelopment poorly understood [12]. Consistent monitoring becomes increasingly critical when a poor repertoire pattern in GMs is frequently observed as it signifies an abnormal GM pattern with a wide range of possible neurodevelopmental alterations [41].

Another aspect to consider is maternal risk factors. This study focused solely on newborn risk factors. Maternal risk factors, such as diabetes in pregnancy, preeclampsia, hypothyroidism, the premature rupture of membranes, abnormal ultrasound findings during pregnancy, maternal drug usage, antepartum hemorrhage, oligohydramnios, chronic medical illness, multiple pregnancies, assisted reproductive techniques, and maternal age, may have varying effects on different areas of MLPT population development [42,43,44].

### Limitations

The limitations of this study include the absence of long-term follow-up for the subjects included in the sample. This is a drawback given the evidence suggesting potential neurodevelopmental challenges during the early preschool years, as alterations in neurodevelopment may manifest later, particularly related to minor neurological disorders. Another important limitation is the small sample size as a larger sample may have yielded statistically significant results for more prevalent risk factors within this population group. This small sample size may also be related to different causes within this research. One of the causes could be a high percentage of not obtaining informed consent since the parents or main caregivers did not sign said document for fear of the potential treatment of the videos. The other cause could be that during the COVID period, there were restrictions on the mobility of the population, preventing researchers from going to hospitals to start the recruitment process. Additionally, there was a limitation in not being able to record the specific amount or intensity of exposure to each risk factor within the assessment tools. This information is crucial for establishing a more precise comparison between these risk factors and GMs.

## 5. Conclusions

Our study found no relationship between GMA and the total scores of the PERI and NBRS in the MLPT population. Additionally, there was no association between the different risk factors and GM patterns.

Within the MLPT population, normal and poor repertoire patterns during the writhing period were most frequently observed.

A direct correlation was identified between the PERI and the NBRS, offering insight into high neurological risk based on risk factors.

There was no correlation between early intervention service attendance and the assessment tools for the early detection of children at high neurological risk.

## Figures and Tables

**Table 1:** Sample Demographics.

Outcome	Moderate-Late Preterm Infants (*n* = 65)
Gestational age (weeks)	34.4 ± 1.27
Weight (g)	2233.96 ± 395.68
Size (cm)	44.11 ± 3.48
Cranial perimeter (cm)	31.54 ± 2.21
Gender (M/F)	36/29
Referred to EI (*n*, %)	13 (20%)

Mean ± standard deviation; cm (centimeters); EI (early intervention); F (female); g (grams); M (male).

**Table 2:** Spearman’s correlation coefficient between Perinatal Risk Inventory and Nursery Neurobiologic Risk Score variables.

		Nursery Neurobiologic Risk Score Items												
		Apgar	Pa0^2^	Ventilation	Blood pH	Apnea + Bradycardia	Hypotension	PDA	Seizures	Intraventricular Hemorrhage	Periventricular Leukomalacia	Infection	Hypoglycemia	Bilirubin
**Perinatal Risk Inventory Items**	**Apgar**	0	0	−0.07	0	−0.06	0	−0.02	0	0	−0.04	−0.05	−0.06	−0.10
	**EEG**	0	0	0	0	0	0	0	0	0	0	0	0	0
	**Seizures**	0	0	0	0	0	0	0	0	0	0	0	0	0
	**Intracranial** **Hemorrhage**	0	0	0.21	0	0.26	0	−0.02	0	1	−0.04	0.29	−0.06	0.16
	**Hydrocephalus**	0	0	0	0	0	0	0	0	0	0	0	0	0
	**Findings CNS**	0	0	0.23	0	0.21	0	0.35	0	−0.04	1	−0.01	0.08	0.05
	**GA**	0	0	0	0	0	0	0	0	0	0	0	0	0
	**Weight**	0	0	−0.15	0	0.05	0	−0.03	0	−0.03	−0.09	0.06	0.04	−0.06
	**Dysmorphic Traits**	0	0	0.40	0	−0.08	0	−0.02	0	−0.02	−0.06	0.42	−0.09	0.23
	**Ventilation**	0	0	0.85	0	0.42	0	−0.08	0	0.17	0.17	0.12	0.07	0.19
	**Head Growth** **(Preterm)**	0	0	0	0	0	0	0	0	0	0	0	0	0
	**Head Growth** **(Aterm)**	0	0	0	0	0	0	0	0	0	0	0	0	0
	**Polycythemia**	0	0	0	0	0	0	0	0	0	0	0	0	0
	**Meningitis**	0	0	0	0	0	0	0	0	0	0	0	0	0
	**Hypoglycemia**	0	0	0.06	0	−0.01	0	0.27	0	−0.08	0.26	0.13	0.82	−0.26
	**Infection**	0	0	0.21	0	−0.08	0	−0.05	0	0.31	−0.14	0.82	0.11	0.43
	**Hyperbilirubinemia**	0	0	0.29	0	0.25	0	−0.09	0	0.17	−0.03	0.33	−0.21	0.87
	**Others**	0	0	0.27	0	0.05	0	0.27	0	−0.06	0.20	0.12	−0.18	0.23

CNS, central nervous system; EEG, electroencephalogram; GA, gestational age; PDA, patent ductus arteriosus.

## Data Availability

Data is contained within the article or Supplementary Materials.

## Supplementary Materials

The following supporting information can be downloaded at https://www.mdpi.com/article/10.3390/jcm12247763/s1: Table S1. Scoring criteria of the Nursery Neurobiologic Risk Score [9]; Table S2. Scoring criteria of the Perinatal Risk Inventory [10].
