# Supplementary material for: The Relationship between General Movements and Risk Factors in Moderate-Late Preterm Infants: A Prospective Cohort Study
Source: J Clin Med. 2023 Dec 18;12(24):7763. doi: 10.3390/jcm12247763 (PMC10743437; doi:10.3390/jcm12247763)
Supplement: Supplementary file 1 [file jcm-12-07763-s001.zip › jcm-2734310-supplementary.pdf]

Journal of
Clinical Medicine

MDPI

Table S1. Scoring criteria of the Nursery Neurobiologic Risk Score [9].

Nursery Neurobiologic Risk Score	
**Item**	**Score**
**Apgar score**	0 = Score > 5 at 1 and 5 min<br>1 = Score ≤ 5 at 1 min and >5 at 5 min<br>2 = Score ≤ 5 at 1 and 5 min<br>4 = Score ≤ 5 at 10 min
**Arterial oxygen pressure (PaO_2)**	0 = PaO_2 never <35 mm Hg<br>1 = PaO_2 < 35 mm Hg for ≤ 2 h (<35 mm Hg ≤ 2 times)<br>2 = PaO_2 < 35 mm Hg for > 2 h (<35 mm Hg > 2 times)<br>4 = PaO_2 ≤ 20 mm Hg for > 2 h (≤20 mm Hg > 2 times)
**Ventilation**	0 = No mechanical ventilation<br>1 = Mechanical ventilation ≤ 7 days<br>2 = Mechanical ventilation 8 to 28 days<br>4 = Mechanical ventilation > 28 days
**Blood pH**	0 = pH never <7.15<br>1 = pH <7.15 for ≤1 h (<7.15 ≤ 2 times) or <7.15 all respiratory, for any duration<br>2 = pH <7.15 for >1 h (<7.15 > 2 times) with metabolic component or pH <7.0 metabolic, for any duration<br>4 = Cardiopulmonary arrest with pH < 7.15
**Apnea with bradycardia**	0 = No apnea or apnea without bradycardia<br>1 = Apnea with bradycardia but without cyanosis ≤20 episodes/day<br>2 = More than 20 episodes of apnea with bradycardia per day, apnea with both bradycardia and cyanosis, or need for manual or mechanical ventilation
**Hypotension**	0 = Systolic blood pressure always >35 mm Hg if infant weighs ≤ 750 gm or >40 mm Hg if infant weighs > 750 gm<br>1 = Hypotension ≤ 2 h (hypotension ≤ 2 times)<br>2 = Hypotension > 2 h (hypotension > 2 times)<br>4 = Hypotension ≥ 6 h
**Patent ductus arteriosus (PDA)**	0 = No PDA<br>1 = PDA responding to fluid restriction, indomethacin, or both<br>2 = PDA in which medical management failed or surgical ligation was required
**Seizures**	0 = No seizures<br>1 = Seizures controlled with one drug and normal interictal electroencephalogram<br>2 = Seizures not controlled with one drug or abnormal interictal electroencephalogram<br>4 = Status epilepticus of ≥12 h
**Intraventricular hemorrhage**	0 = No hemorrhage<br>1 = Subependymal germinal matrix hemorrhage only<br>2 = Blood in one or both ventricles<br>4 = Intraparenchymal blood or development of overt hydrocephalus
**Periventricular leukomalacia**	0 = No periventricular leukomalacia<br>1 = Questionable changes followed by return to normal state

	2 = Moderate (definite) changes followed by return to normal state<br>4 = Cyst formation or cerebral atrophy with large ventricles
**Infection**	0 = No infection or antibiotics given for possibility of infection with negative cultures<br>1 = Infection highly suspected or documented without changes in blood pressure<br>2 = Septic shock (documented sepsis and hypotension)<br>4 = Meningitis
**Hypoglycemia**	0 = No glucose level <30 mg/dL (1.7 mmol/L)<br>1 = Glucose level <30 mg/dL, asymptomatic, and ≤6 hours' duration<br>2 = Glucose level <30 mg/dL, asymptomatic for >6 h or symptomatic for any duration<br>4 = Glucose level <30 mg/dL for >24 h and symptomatic
**Bilirubin**	0 = Bilirubin concentration ≤10 mg/dL if ≤1 kg birth weight or ≤0.01 X birth weight in grams if >1 kg<br>1 = Bilirubin concentration >10 mg/dL if ≤1 kg birth weight or >0.01 X birth weight in grams if >1 kg<br>2 = Exchange transfusion for hyperbilirubinemia

Table S2. Scoring criteria of the Perinatal Risk Inventory [10].

**Perinatal Risk Inventory**	
**Item**	**Score**
**Apgar score**	0 = No neurobehavioral abnormalities<br>1 = Hyperalert<br>2 = Mild hypotonia<br>3 = Severe hypotonia
**Electroencephalogram**	0 = Normal electroencephalogram or not performed<br>1 = Abnormal electroencephalogram but normal at discharge<br>2 = Abnormalities on electroencephalogram (not flat or periodic) with continued abnormalities at discharge<br>3 = Periodic or flat electroencephalogram with continued abnormalities at discharge
**Seizures (nonmetabolic)**	0 = No problem<br>1 = Suspected seizure not treated with anticonvulsants<br>2 = One or more seizures with response to a single anticonvulsant<br>3 = One or more seizures with resistance to therapy requiring two or more anticonvulsants
**Intracranial hemorrhage**	0 = Negative computed tomography or ultrasound or not performed<br>1 = Subarachnoid hemorrhage with seizures or grade I and grade II intraventricular hemorrhage<br>2 = Grade III intraventricular hemorrhage<br>3 = Grade IV intraventricular hemorrhage
**Hydrocephalus**	0 = No evidence of hydrocephalus on ultrasound or computed tomography<br>1 = Suspected on clinical basis, resolved without treatment

	2 = Hydrocephalus confirmed on ultrasound or computed tomography, without shunt, treated medically or repeated intraventricular taps<br>3 = Hydrocephalus confirmed on computed tomography or ultrasound, shunt required
**Computed tomography or ultrasound without evidence of hydrocephalus or intracranial hemorrhage, but other central nervous system findings**	0 = Negative computed tomography or ultrasound or not done<br>1 = Abnormal findings with return to normal prior to discharge<br>2 = Identified abnormalities not specified elsewhere without return to normal prior to discharge<br>3 = Loss of parenchyma, decreased mantel, or other abnormalities that may result in parenchymal injury not specified elsewhere
**Premature with weight >3rd percentile (appropriate for gestational age)**	0 = >32 weeks<br>1 = 32–30 weeks<br>2 = 29–27 weeks<br>3 = 26–24 weeks
**Weight for gestational age**	0 = Weight appropriate for gestational age<br>1 = <10th percentile for weight but >3rd percentile<br>2 = <3rd percentile for weight<br>3 = <3rd percentile for weight, with a 2 or 3 in other categories of perinatal index
**Dysmorphic features**	0 = None or one dysmorphic feature<br>1 = Two minor dysmorphic features<br>2 = Three or more minor dysmorphic features or one major feature with normal chromosomes<br>3 = Chromosomal abnormalities or a syndrome known to be associated with developmental disabilities such as Down syndrome or fetal alcohol syndrome
**Ventilation**	0 = Not ventilated<br>1 = 7 days or less<br>2 = 8 to 21 days<br>3 = >21 days or clinical diagnosis of bronchopulmonary dysplasia with tachypnea
**Head growth (premature infant hospitalized 6 weeks or more)**	0 = Head size >10% and <90% for gestational age with 3.5 cm or greater growth in first 6 weeks<br>1 = Initial head circumference in the 5th-10th percentile with 3.5 cm or greater growth in the first 6 weeks<br>2 = Initial head circumference in >3rd percentile with less than 3.5 cm growth in the first 6 weeks<br>3 = Initial head circumference <3rd percentile for gestational age with <3.5 cm for the first 6 weeks
**Head growth (term infants hospitalized >3 weeks)**	0 = Initial head circumference > 10th percentile with average head growth ≥0.3 cm/wk<br>1 = Initial head circumference > 10th percentile with average head growth <0.29 cm/wk<br>2 = Initial head circumference below the 10th percentile with average head growth >0.3 cm/wk<br>3 = Initial head circumference below the 10th percentile with average head growth <0.29 cm/wk

**Polycythemia**	0 = Hematocrit <65%<br>1 = Hematocrit >65% and <70% without exchange transfusion<br>2 = Hematocrit >65% without symptoms with exchange transfusion<br>3 = Hematocrit >65% requiring exchange transfusion because of hypoglycemia, lethargy, apnea, or seizures
**Meningitis**	0 = None<br>1 = Suspected diagnosis on clinical or laboratory basis without bacterial or viral confirmation<br>2 = Confirmed diagnosis with or without seizures with adequate tone and state control within 72 h<br>3 = Confirmed diagnosis with persistent hypotonia or obtunded state or seizures that persist for more than 72 h
**Hypoglycemia**	0 = No hypoglycemia<br>1 = Hypoglycemia without symptoms requiring oral feedings only<br>2 = Hypoglycemia with lethargy or hypotonia requiring treatment with IV glucose<br>3 = Hypoglycemia with seizures requiring treatment with IV glucose, glucagon, or corticotropin
**Congenital infection**	0 = No suspicion of congenital infection<br>1 = Suspected, but without viral or serologic confirmation<br>2 = Suspected, may include small for gestational age only with viral or serologic confirmation<br>3 = Clearly identified diagnosis by culture or serology associated with signs and symptoms, ie., jaundice, chorioretinitis, or hepatosplenomegaly
**Hyperbilirubinemia**	0 = Not requiring therapy<br>1 = Mild, requiring phototherapy or single exchange<br>2 = Hyperbilirubinemia requiring two or more exchange transfusions<br>3 = Hyperbilirubinemia requiring two or more exchange transfusions and associated with neurologic changes such as lethargy or increased irritability
**Associated medical problems such as hydrops, retinopathy of prematurity, cyanotic heart disease, bronchopulmonary dysplasia, and necrotizing enterocolitis (non-central nervous system)**	0 = No associated medical problems complicating the neonatal course<br>1 = Associated medical problems suspected but not substantiated<br>2 = Established neonatal problems, but resolved prior to discharge<br>3 = Persistent medical problems at time of discharge such as grade III retinopathy of prematurity, ileostomy, supplemental oxygen requirement, or nasogastric tube
